# Household Transmission and Clinical Features of Respiratory Tract Infections That Were SARS-CoV-2 Positive and Negative

**DOI:** 10.1093/infdis/jiae278

**Published:** 2024-05-31

**Authors:** Jaakko Ahti, Laura Toivonen, Helena Ollila, Lauri Ivaska, Krista Salo-Tuominen, Tytti Vuorinen, Johanna Lempainen, Ville Peltola

**Affiliations:** Department of Paediatrics and Adolescent Medicine, Turku University Hospital and University of Turku; Department of Paediatrics and Adolescent Medicine, Turku University Hospital and University of Turku; Department of Biostatistics, Turku University Hospital and University of Turku; Department of Paediatrics and Adolescent Medicine, Turku University Hospital and University of Turku; InFLAMES Flagship, University of Turku; Department of Paediatrics and Adolescent Medicine, Turku University Hospital and University of Turku; Unit of Health and Well-Being, Turku University of Applied Sciences; Department of Clinical Microbiology, Turku University Hospital; Institute of Biomedicine, University of Turku; Department of Paediatrics and Adolescent Medicine, Turku University Hospital and University of Turku; Immunogenetics Laboratory, Institute of Biomedicine, University of Turku, Finland; Department of Paediatrics and Adolescent Medicine, Turku University Hospital and University of Turku; InFLAMES Flagship, University of Turku

**Keywords:** children, COVID-19, household transmission, SARS-CoV-2, secondary attack rate

## Abstract

**Background:**

Comparative data are limited on the transmission of respiratory infections positive and negative for SARS-CoV-2 in households with children.

**Methods:**

In June to August 2020, we recruited 700 participants (175 households, 376 children, 324 adults) to be prospectively followed for all respiratory tract infections. Follow-up lasted from recruitment until April 2022. Daily symptoms were monitored by weekly electronic questionnaires. SARS-CoV-2 polymerase chain reaction testing from nasopharyngeal specimens was performed for symptomatic participants and twice (1-week interval) for the household members of positive participants. Clinical features and secondary attack rates (SARs) based on the onset of symptoms were compared between respiratory infections that were SARS-CoV-2 positive and negative.

**Results:**

Most SARS-CoV-2 infections (90%) occurred from January to April 2022 when Omicron BA.1 and BA.2 were the dominant variants. SARS-CoV-2–positive infections were transmitted more often than SARS-CoV-2–negative infections (SAR, 41% vs 24%; *P* < .001). SARS-CoV-2 transmission was similar for child and adult index cases (SAR, 40% vs 43%; *P* = .47), but the transmission of SARS-CoV-2–negative infections was higher for child index cases (SAR, 27% vs 18%; *P* < .001).

**Conclusions:**

Our findings demonstrate that SARS-CoV-2 Omicron viruses spread more effectively within households as compared with other respiratory infections.

## Teaser

In a Finnish cohort, household transmission of SARS-CoV-2 infections was higher when compared with SARS-CoV-2–negative respiratory infections. SARS-CoV-2 transmission was similar for adult and child index cases, whereas the transmission of SARS-CoV-2–negative infections was higher for child index cases.

## Graphical abstract

**Graphical abstract:**
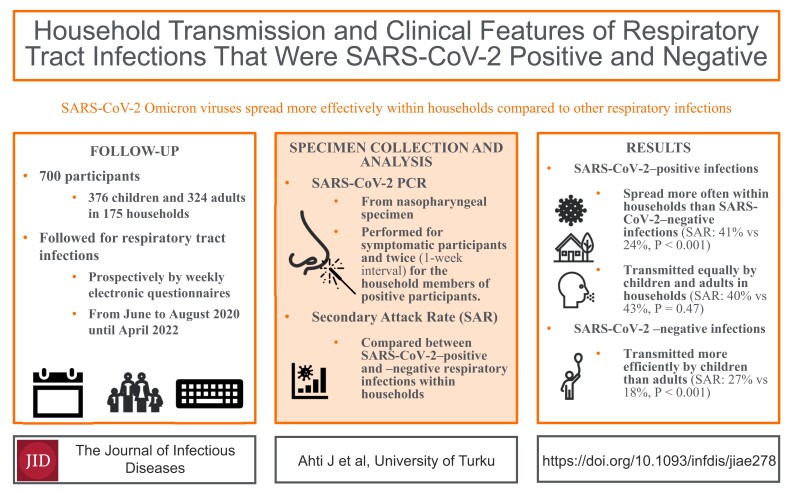

During the COVID-19 pandemic, preventive measures were applied to limit the transmission of SARS-CoV-2 in communities worldwide [1–3]. The means of prevention within households are limited, leading to households being one of the main sources of SARS-CoV-2 transmission [4–9]. Secondary attack rates (SARs) in households for Delta and Omicron variants of SARS-CoV-2 have been reported to be higher than those for pre-Delta variants of SARS-CoV-2 [10–15].

Children play an important role in the transmission of respiratory infections in communities [16–18]; however, for the pre-Delta variants of SARS-CoV-2, children have been reported to transmit SARS-CoV-2 to contact subjects more seldom than adults [19–21]. More recently emerged variants of SARS-CoV-2 may have different transmission dynamics. Several studies have reported that children's susceptibility to SARS-CoV-2 infection and the probability to transmit the disease further have increased after the emergence of Delta and Omicron variants as compared with pre-Delta variants of SARS-CoV-2 [10–13, 22–24].

Understanding the dynamics of SARS-CoV-2 transmission in households is essential to further develop preventive measures and minimize the negative effects of restrictions on children [25–27]. This study aimed to examine the transmission and clinical presentation of SARS-CoV-2–positive and SARS-CoV-2–negative infections in households with children.

## METHODS

### Study Population

The current research was conducted within an observational prospective birth cohort study called the STEPS Study (Steps to the Healthy Development and Well-being of Children). In the STEPS Study, 1805 children born in 2008 to 2010 in the Hospital District of Southwest Finland are systematically followed from pregnancy to early adulthood [28]. Of these children, 175 children and all their household members (a total of 702 participants) volunteered to participate in this study in June to August 2020. The study protocol was approved by the Ethics Committee of the Hospital District of Southwest Finland. Adult and adolescent participants provided written informed consent before enrollment. Parental written consent was obtained for all participants <18 years old.

### Follow-up

The participants were monitored for daily symptoms, SARS-CoV-2 polymerase chain reaction (PCR) and home antigen testing and results, and treatment of respiratory infections through weekly REDCap [29, 30] questionnaires from recruitment to the end of the study in April 2022. The detailed contents of the questionnaires are reported in the supplementary methods. In addition to the weekly questionnaires, the results of the SARS-CoV-2 PCR tests and SARS-CoV-2–related hospital admissions and medications were collected from the electronic medical records of the Hospital District of Southwest Finland. Data on COVID-19 vaccinations of participants were obtained by separate questionnaires. Participants who received ≥2 COVID-19 vaccine doses were considered fully vaccinated. For analysis by vaccination status, the COVID-19 vaccination was considered effective 14 days after the vaccination date.

### Specimen Collection and Analysis

Throughout the study, all participants exhibiting symptoms associated with SARS-CoV-2 infection and those exposed to the virus were referred to nasopharyngeal swab specimen collection by health care personnel at local SARS-CoV-2 testing stations. This practice was in accordance with the Finnish national guidelines during the COVID-19 pandemic (supplementary methods). Starting January 2022, the national guideline was changed to allow the use of home antigen tests as an alternative to laboratory testing. The study participants were still encouraged to undergo nasopharyngeal specimen collection by health care personnel for SARS-CoV-2 PCR, but use of home antigen tests was also allowed. If a study participant tested positive for SARS-CoV-2, we referred all other household members to specimen collection for SARS-CoV-2 PCR on the same or following day and 1 week after the initial test. If any household member developed respiratory symptoms later, the concerned participant was subjected to an additional SARS-CoV-2 PCR test at the onset of the symptoms. Specimens were analyzed by a laboratory-developed real-time reverse transcriptase–PCR for detection of SARS-CoV-2 at the Department of Clinical Microbiology, Turku University Hospital [31]. The test detected 2 targets in SARS-CoV-2: E gene by using World Health Organization–recommended primers and probe (the diagnostic target) and S gene based on laboratory-designed primers and 2 competing probes that distinguish whether there is an H69-V70 deletion in the S gene sequence. The SARS-CoV-2 variant determination was performed by sequencing on a convenience sample of the positive specimens depending on circulation of SARS-CoV-2 variants of concern. Data on circulating SARS-CoV-2 variants in Southwest Finland based on routine surveillance were obtained from the Department of Clinical Microbiology at the Turku University Hospital, and periods of dominant virus variants were determined.

### Definitions and Outcome Measures

Participants were defined as adults or children by their role as parents or offspring in the household, regardless of age. An index case was defined as the first household member identifying symptoms compatible with COVID-19 or another acute respiratory tract infection. An acute respiratory infection was defined as an acute onset of cough, rhinorrhea, or sore throat, with or without fever. A respiratory infection cluster was defined to include all respiratory infections in a household with the onset of symptoms within a 14-day period from the onset of symptoms in the index case. All nonindex household members identifying symptoms of infection were considered secondary cases. Respiratory infection clusters with an unidentifiable index person and clusters with no SARS-CoV-2 test performed for any symptomatic household member were excluded from the analysis. The flow of participants and households in the study is shown in Supplementary Figure 1, and the flow of cluster inclusion is shown in Supplementary Figure 2.

The respiratory infection cluster was considered SARS-CoV-2 positive if any household member tested positive for SARS-CoV-2. A cluster was considered SARS-CoV-2 negative if at least 1 household member provided a negative SARS-CoV-2 PCR test result during the current infection cluster and none of the tests were SARS-CoV-2 positive. Asymptomatic individual infections (n = 7) were not included in the clusters, regardless of their SARS-CoV-2 status. Symptomatic individuals who provided negative SARS-CoV-2 test results during a SARS-CoV-2–positive cluster were not included in SARS-CoV-2–positive clusters. Transmission in households was evaluated by using the household SAR as the main outcome. SAR was defined as the number of nonindex household members presenting symptoms of infection within 14 days of the onset of symptoms of the index case, divided by the total number of nonindex household members. Other outcomes included serial intervals in households (time from the onset of symptoms in the index case to that in a secondary case) and clinical characteristics of infections.

### Statistical Analysis

The chi-square test was used to compare the demographic factors between children and adults. Asymptomatic SARS-CoV-2–positive infections (n = 7) were excluded from calculations of secondary transmission, serial intervals, SAR, and comparison of clinical features. The symptoms between SARS-CoV-2–positive and SARS-CoV-2–negative infections were examined by mixed effects logistic regression. In the models, the SARS-CoV-2 test result was treated as a fixed effect, and participant identity and household identity were treated as random effects. While identity variables were introduced as a random effect, the repetition of individuals and households in the data was considered. Natural logarithmic transformation was performed for the durations of all symptoms. The estimated duration of symptoms for infections that were SARS-CoV-2 positive and negative was examined via a linear mixed model. The same effects were used as with the prevalence models. The differences between children and adults were examined with the same tests but used the household role (child vs adult) as a fixed effect.

Natural logarithmic transformation was performed for the interval between the date of the onset of symptoms of the index patient and that of the household contact due to nonnormal distribution. The statistical difference between SARS-CoV-2–positive and SARS-CoV-2–negative infection clusters was analyzed by a linear mixed model. The SARS-CoV-2 positivity of the cluster was treated as a fixed effect and household identity as a random effect in the model.

The infection clusters of the individuals were treated independently. Statistical differences between SARS-CoV-2–positive and SARS-CoV-2–negative infections and associations between SARs and household and index case characteristics were analyzed with a Wald risk difference test. Characteristics included household size (1 or 2 vs ≥3 children), index case role (child vs adult), and number of COVID-19 vaccinations per index case (0 or 1 vs 2 or 3). The level of significance was set at *P* < .05 (2-tailed). The statistical analyses were performed with SAS version 9.4 (SAS Institute).

## RESULTS

### Characteristics of Study Participants

**Figure 1.**
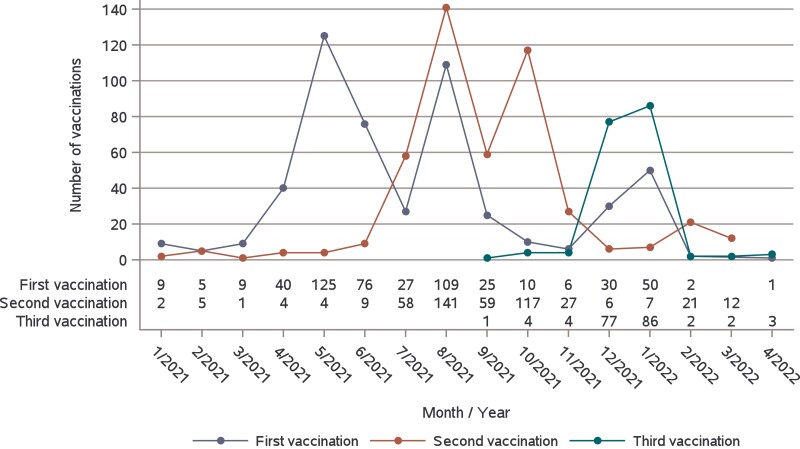
Progression of COVID-19 vaccinations by month in the study population.

We recruited 702 participants from 175 households from June to August 2020 (Supplementary Figure 1). Two were excluded after recruitment. Altogether, 624 participants and 155 households completed the study until the end of April 2022, and 76 participants (20 households) dropped out. Of the 700 participants included in the analysis, 376 (54%) were children (median age, 11.1 years) and 324 (46%) were adults (median age, 43.5 years; Table 1). The age range was 0.3 to 62 years. Nine children were ≥18 years of age but were defined as children by their role in the household. Of the households, 48 (27%) had ≥3 children and were considered large households. At the end of the study, 224 (60%) children and 274 adults (85%) had received ≥2 doses of the COVID-19 vaccine. The progression of vaccinations in the study participants is shown in Figure 1.

**Table 1.** Demographic and Clinical Characteristics of 700 Participants in 175 Households

Characteristic	All (N = 700)	Children (n = 376)	Adults (n = 324)	*P* Value^a^
Sex				
Male	344 (49)	195 (52)	149 (46)	…
Female	356 (51)	181 (48)	175 (54)	.12
Age, y, median (IQR)	15.8 (10.9–43.2)	11.1 (9.4–12.1)	43.5 (40.1–47.6)	…
No. of COVID-19 vaccines received^b^				<.0001
0	66 (9)	53 (14)	13 (4)	
1	57 (8)	50 (13)	7 (2)	
2 or 3	498 (71)	224 (60)	274 (85)	
Unknown	79 (11)	49 (13)	30 (9)	
SARS-CoV-2 infections^c^				
Whole study period	334 (48)	185 (49)	149 (46)	.40
1 Jun 2020–31 Dec 2021	34 (10)	22 (12)	12 (8)	…
1 Jan 2022–29 Apr 2022	300 (90)	163 (88)	137 (92)	.25

Data are presented as No. (%) unless otherwise stated.

^a^Comparison between children and adults by chi-square test.

^b^By 29 April 2022.

^c^The number of participants infected at least once. Eight participants were infected twice, and the total number of SARS-CoV-2–positive infections was 342.

### Epidemiology of Infections: SARS-CoV-2 Positive and Negative

**Figure 2.**
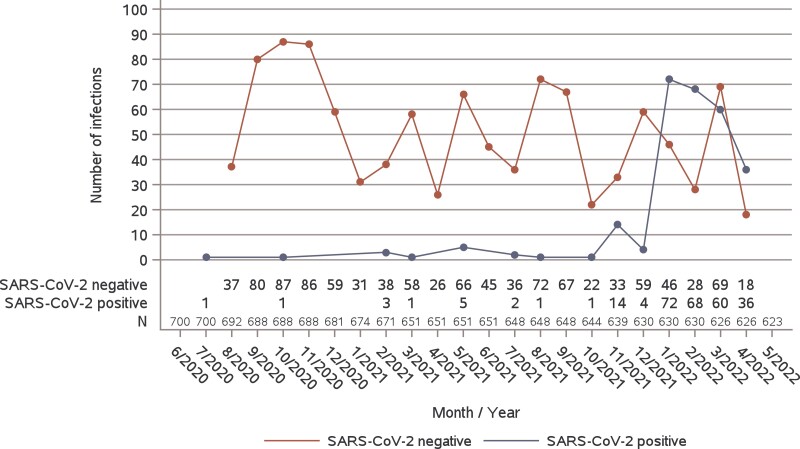
Incidence of infections during the study that were SARS-CoV-2 positive and negative. The number of individuals in the follow-up are shown monthly.

We analyzed 728 respiratory infection clusters in 150 households (Supplementary Figure 2). Household respiratory infection clusters consisted of 1332 separate acute respiratory infections documented in 489 individuals. Of 728 household respiratory infection clusters, 120 (16%) were SARS-CoV-2 positive and 608 (84%) were SARS-CoV-2 negative. SARS-CoV-2–positive clusters (n = 120) consisted of 262 individual symptomatic infections. Among them, 252 (96%) tested positive for SARS-CoV-2, while 10 (4%) were not tested but were included in positive clusters. SARS-CoV-2–negative clusters (n = 608) comprised 1063 individual symptomatic infections, of which 839 (79%) tested negative for SARS-CoV-2. Of 1091 total individual tests conducted in clusters that were SARS-CoV-2 positive and negative, 934 (86%) were SARS-CoV-2 PCR tests while 157 (14%) were home antigen tests. Most SARS-CoV-2–positive infections occurred in 2022 (Table 1, Figure 2), with Omicron BA.1 as a dominant variant and BA.2 emerging from week 5 onward in the study region (Supplementary Table 1). SARS-CoV-2–negative infections were observed constantly during the study. No significant difference was observed in the proportions of SARS-CoV-2–positive and SARS-CoV-2–negative infections between adults and children. Respiratory virus circulation in Southwest Finland during the study period is shown in Supplementary Figure 3.

### Clinical Characteristics of Respiratory Infections

All documented SARS-CoV-2 infections in adults and 97.4% of those in children were symptomatic. Fever and cough were more common symptoms during SARS-CoV-2–positive infections vs SARS-CoV-2–negative infections in children and adults (all *P* values ≤.01; Table 2). Rhinorrhea was less common during SARS-CoV-2–positive than SARS-CoV-2–negative infections in children (58% vs 72%, *P* = .003). No significant difference was noted in the duration of any symptom. Further comparisons of symptoms between SARS-CoV-2–positive and SARS-CoV-2–negative infections and between children and adults are shown in Supplementary Tables 2 and 3. One adult participant died due to COVID-19. No other participant was hospitalized for COVID-19 during the study.

**Table 2.** Symptom Prevalence and Duration of Infections in Children and Adults Who Were SARS-CoV-2 Positive and Negative

	Children				Adults			
Symptom	Positive (n = 149)^a^	Negative (n = 656)	*P* Value^b^	OR (95% CI)^c^	Positive (n = 120)	Negative (n = 407)	*P* Value^b^	OR (95% CI)^c^
Fever								
Prevalence	73 (51)	135 (21)	<.0001	6.06 (3.85–9.54)	61 (51)	60 (15)	<.0001	6.82 (4.05–11.49)
Duration, d	3 (2–3)	2 (2–3)	.11	…	3 (2–4)	2 (2–3)	.42	…
Cough								
Prevalence	83 (58)	294 (45)	.0010	1.67 (1.13–2.48)	91 (76)	156 (38)	<.0001	5.30 (3.22–8.74)
Duration, d	4 (3–7)	5 (3–7)	.82	…	5 (4–9)	5 (4–8)	.65	…
Rhinorrhea								
Prevalence	83 (58)	471 (72)	.0033	0.56 (.38–.82)	96 (80)	299 (73)	.13	1.48 (.88–2.48)
Duration, d	5 (3–6.5)	5 (4–7)	.99	…	5 (3–7.5)	5 (4–7)	.66	…
Sore throat								
Prevalence	93 (65)	445 (68)	.76	0.94 (.61–1.43)	83 (69)	267 (66)	.42	1.21 (.76–1.91)
Duration, d	4 (3–6)	3 (3–5)	.066	…	3 (3–5)	3 (2–5)	.30	…

Data are presented as No. (%) or median (IQR).

Abbreviation: OR, odds ratio.

^a^Asymptomatic SARS-CoV-2–positive infections were excluded (n = 7, all in children).

^b^Symptom prevalence was compared by mixed effects logistic regression and duration by a linear mixed model.

^c^SARS-CoV-2–positive infections vs SARS-CoV-2–negative infections.

### Household Transmission

**Figure 3.**
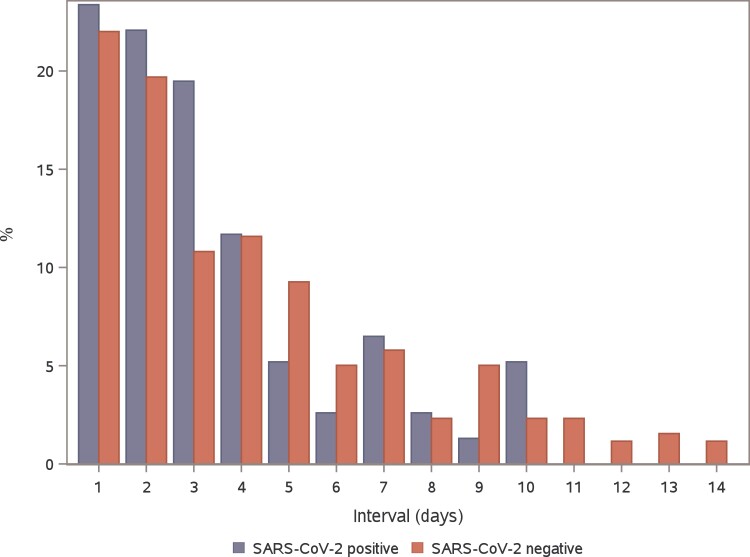
Distributions of serial intervals in household clusters that were SARS-CoV-2 positive and negative.

The median serial interval was 3 days in clusters that were SARS-CoV-2 positive (IQR, 2–4 days) and SARS-CoV-2 negative (IQR, 2–6 days; *P* = .15; Figure 3). Secondary transmission occurred in 70.8% of SARS-CoV-2–positive household clusters (95% CI, 62.7%–79.0%) and 46.4% of SARS-CoV-2–negative household clusters (95% CI, 42.4%–50.4%; *P* < .001). The overall SARs were 41.4% (95% CI, 36.4%–46.5%) and 24.3% (95% CI, 22.3%–26.2%) for clusters that were SARS-CoV-2 positive and negative, respectively (*P* < .001). In SARS-CoV-2–positive clusters, 52% (59/114) of index cases and 54% (83/155) of secondary cases were children, whereas in SARS-CoV-2–negative clusters, children accounted for 69% (424/614) and 52% (232/449), respectively. At the symptom onset in SARS-CoV-2–positive clusters, 59% (66/111) of index persons and 69% (102/148) of household members were fully vaccinated against COVID-19.

**Figure 4.**
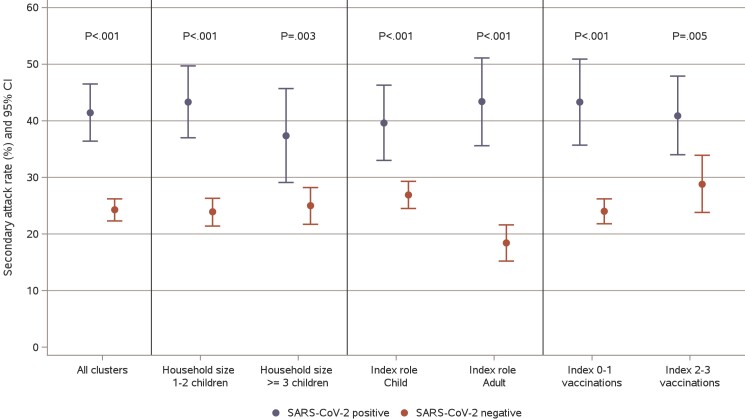
Secondary attack rates (SARs) of SARS-CoV-2–positive and SARS-CoV-2–negative infections assessed by household and index case characteristics. *P* values are shown for comparisons of SARs between clusters with respiratory infection that were SARS-CoV-2 positive and negative. Within SARS-CoV-2–positive clusters, there were no significant differences in SARs by household and index case characteristics. Within SARS-CoV-2–negative clusters, the SAR was significantly lower when the index case was a child (*P* < .001); other characteristics were not significantly associated with it.

Household transmission of SARS-CoV-2–positive and SARS-CoV-2–negative clusters by household and index case characteristics is shown in Figure 4. In all compared groups, the SAR was significantly higher for SARS-CoV-2–positive clusters than for SARS-CoV-2–negative clusters. The effect of household size and index case characteristics (role and vaccinations) for transmission were analyzed separately in clusters that were SARS-CoV-2 positive and negative. In SARS-CoV-2–positive clusters, the SAR for the child index case was 39.6% (95% CI, 33.0%–46.3%) and the SAR for the adult index case was 43.4% (95% CI, 35.6%–51.1%; *P* = .47). The SAR for the child secondary case was 42.6% (95% CI, 35.6%–49.5%) and the SAR for the adult secondary case was 39.2% (95% CI, 31.9%–46.5%; *P* = .51). No significant difference in SAR was found for any assessed factor for SARS-CoV-2–positive clusters. In SARS-CoV-2–negative clusters, the SAR for the child index case was significantly higher (26.9%; 95% CI, 24.5%–29.3%) than that for the adult index case (18.4%; 95% CI, 15.2%–21.6%; *P* < .001). The SAR for the child secondary case was 25.7% (95% CI, 22.9%–28.6%) and that for the adult secondary case was 22.8% (95% CI, 20.1%–25.5%; *P* = .14).

## DISCUSSION

In this study, we investigated the roles of children and adults in the household transmission of SARS-CoV-2 as compared with other respiratory tract infections during the same study period. There was no difference between children and adults in SARS-CoV-2 transmission rates in households. In contrast, we found that children were significantly more efficient in transmitting SARS-CoV-2–negative infections onward as compared with adults.

Most SARS-CoV-2 infections in our study occurred in 2022 when the Omicron BA.1 and BA.2 variants were dominant, matching the epidemiology of the COVID-19 pandemic in Finland with a low national incidence of SARS-CoV-2 until the beginning of 2022 (see supplementary appendix for national COVID-19 vaccination policies, testing strategies, and nonpharmaceutical preventive measures) [32]. Consequently, most SARS-CoV-2 infections in our study can be suggested to be primary infections in persons with no prior natural immunity against the pandemic virus. On the contrary, SARS-CoV-2–negative infections were found throughout the study period, which aligns with regional surveillance data and reported ongoing rhinovirus circulation in Finland during the pandemic (Supplementary Figure 3) [33].

The SAR for SARS-CoV-2–positive infections (42%) in our study is in line with a meta-analysis regarding the Omicron variant (mean SAR, 43%; 95% CI, 35%–50%) but higher when compared with a meta-analysis of pre-Omicron variants [10]. A household cohort study conducted in Egypt in 2020 reported a SAR of 90% [5], and a household study done in Finland in 2020 reported a SAR of 45% [34]. Both of these studies included seropositive cases. These results indicate that the SAR for SARS-CoV-2 varies considerably among study populations, settings, and virus variants but is high during Omicron circulation.

In our study, the household transmission of SARS-CoV-2 was efficient even though 85% of adults were fully vaccinated against COVID-19. Additionally, the majority of the children and adolescents (60%) received ≥2 doses of the vaccine by the end of the study, but vaccinations of children aged 5 to 11 years started only in December 2021, at the start of the Omicron wave. We found no difference in SARs between partly and fully vaccinated participants. These findings support the previously reported evidence of low vaccine effectiveness against Omicron transmission [10, 14, 35].

The role of children in the household transmission of SARS-CoV-2 has increased from the pre-Delta variants to the Omicron variant. A meta-analysis found that child contacts had a lower household SAR as compared with adult contacts of SARS-CoV-2 pre-Delta variants, but this difference was not significant for the Delta and Omicron variants [13]. The study also reported a household SAR of 56% for the Omicron variant and child secondary contacts. We found a slightly lower household SAR for the child secondary case (44%). Notably, children in our study were older (median age, 11 years) than in most other studies. Generally, young children have an important role in the spread of respiratory viruses [16].

Importantly, this study adds to the literature by comparing the household transmission of SARS-CoV-2–positive and SARS-CoV-2–negative infections within a Finnish cohort. We found that the household SAR was higher for infections that were SARS-CoV-2 positive than negative and that the SAR for SARS-CoV-2 was independent of the index case's vaccination status, household role (child vs adult), and household size. Our findings indicate that, even in highly vaccinated populations, adults and children efficiently spread SARS-CoV-2 Omicron variants, in contrast to the more dominant role of children in the transmission of other respiratory viruses. Earlier studies have reported household SARs for some non–SARS-CoV-2 respiratory viruses. The SARs for the 2009 pandemic influenza A (H1N1) virus were 23% and 51% in studies performed at summer camps [36, 37], whereas in 2 studies of seasonal influenza, the SARs were 6% and 31%, with children having significantly higher SAR values than adults in both studies [38, 39]. For rhinovirus, higher rates of transmission were observed from child index cases to siblings (100% rhinovirus positive) than parents (50% rhinovirus positive) [40]. For respiratory viruses in general, Schlinkmann et al reported a secondary attack proportion of 0.2 [16], which is similar to what we found for SARS-CoV-2–negative infections. Schlinkmann et al also reported an elevated risk for transmission for children aged 0 to 6 years. Correspondingly in our study, the household SAR for SARS-CoV-2–negative child index cases was higher when compared with adult index cases, even though the school-age children in our study may be weaker transmitters of viruses than younger children.

We determined that children and adults with SARS-CoV-2 presented more often with fever and cough than those with SARS-CoV-2–negative infections, who presented with rhinorrhea and sore throat as the most frequent symptoms. The prevalence of respiratory symptoms and resolution of acute symptoms of SARS-CoV-2 infections in our study were similar to those reported by Menni et al in their large observational study of the Omicron variant [35]. Of interest, we detected only 7 (2.6%) asymptomatic SARS-CoV-2 infections, all in children. A recent review reported that asymptomatic infections were more common in children and adolescents than in adults for the Omicron variant, with a corresponding proportion of asymptomatic infections of 26% (95% CI, 17%–38%) [41]. Reasons for the low number of asymptomatic infections in our study may include detailed daily documentation of even mild symptoms and other features of the study design.

Our study has several strengths. We prospectively followed for a long time households that were recruited from an ongoing unselected birth cohort study. In addition to weekly questionnaires, data were collected from electronic medical records, which added to the reliability of the results. To the best of our knowledge, this is among the first studies to present a comparison of SARS-CoV-2 household transmission to respiratory infections that were negative for SARS-CoV-2. However, we also note a few limitations of our study. First, even though the follow-up rate was considerably good, we noticed slightly decreased response rates toward the end of the study. Decreased willingness to participate might have also led participants to evade testing while symptomatic. Second, the causative agents for the SARS-CoV-2–negative clusters were not specified; positive results of home antigen tests were accepted; all secondary cases were not studied for viruses; and the recorded symptoms were nonspecific. These features of the study might have led to misclassification of clusters or individual infections. Yet, the majority of secondary infections in positive and negative clusters were tested for SARS-CoV-2 by PCR with a similar result as the index case. Also, a temporal difference in the occurrence of clusters that were SARS-CoV-2 negative (throughout the study period) and positive (mostly in 2022), extensive use of SARS-CoV-2 PCR tests as compared with antigen tests, and short periods of clusters (14 days) support correct classification. Nevertheless, our results should be interpreted as conservative, since if some misclassification occurred, it has decreased the observed differences between the SARS-CoV-2–negative and SARS-CoV-2–positive clusters. Third, the role of natural immunity was not investigated in the study. Fourth, although the study period was almost 2 years, most COVID-19 cases were dated in 2022 due to the comparatively low incidence of SARS-CoV-2 in Finland before Omicron. Consequently, we acquired sufficient data for only the Omicron variant of SARS-CoV-2. Fifth, as daily symptoms were monitored only once a week, recall bias is possible. Finally, as the SAR of respiratory infections varies considerably among study populations, settings, and virus variants, the generalizability of our results may be limited.

In conclusion, in this prospective study, we discovered that SARS-CoV-2–positive infections were transmitted in households more efficiently than SARS-CoV-2–negative infections. Furthermore, there were no differences between children and adults in either SARS-CoV-2 transmission or COVID-19 susceptibility, although children transmitted SARS-CoV-2–negative infections more efficiently than adults. Our results corroborate the perception that SARS-CoV-2 Omicron viruses are particularly efficient in spreading in households, despite good vaccination coverage. What is different from other respiratory infections is that adults and children are important in household transmission of SARS-CoV-2.

## Supplementary Data

Supplementary materials are available at *The Journal of Infectious Diseases* online (http://jid.oxfordjournals.org/). Supplementary materials consist of data provided by the author that are published to benefit the reader. The posted materials are not copyedited. The contents of all supplementary data are the sole responsibility of the authors. Questions or messages regarding errors should be addressed to the author.

## Supplementary Material

jiae278_Supplementary_Data

## Notes

***Acknowledgments.*** Ulla Torkko and Kati Pyykkö are thanked for their assistance in recruiting the participants and for their help with conducting the study. Joe Hettinger is thanked for the language revision.

***Author contributions.*** Conceptualization of trial: J. A., L. T., L. I., K. S.-T., and V. P. Funding acquisition: J. A., L. T., and V. P. Supervision: L. T. and V. P. Project administration: J. A., L. T., L. I., and V. P. Investigation, data curation, and resources: J. A., L. T., H. O., L. I., K. S.-T., T. V., J. L., and V. P. Interpretation of data: J. A., L. T., H. O., and V. P. Formal analysis and visualization: J. A., L. T., H. O., and V. P. Drafting of manuscript: J. A., L. T., H. O., and V. P. All authors reviewed and edited the draft manuscript, approved the final version, agreed to be accountable for all aspects of the published work, and are responsible to appropriately resolve and investigate all questions related to the accuracy or integrity of the published work.

***Data sharing.*** Data are not publicly available. This study did not entail large data sets that are routinely deposited in online repositories.

***Financial support*.** This work was supported by research funds from Specified Government Transfers and Hospital District of Southwest Finland; and by grants to J. A. from the Government Research Foundation of the Satakunta Hospital District, the Paulo Foundation, the Päivikki and Sakari Sohlberg Foundation, and the Maud Kuistila Foundation.
