# Supplementary material for: Household Transmission and Clinical Features of Respiratory Tract Infections That Were SARS-CoV-2 Positive and Negative
Source: J Infect Dis. 2024 May 31;230(4):e837–46. doi: 10.1093/infdis/jiae278 (PMC11481349; doi:10.1093/infdis/jiae278)
Supplement: jiae278_Supplementary_Data [file jiae278_supplementary_data.docx]

**Supplementary Appendix**

**Household Transmission and Clinical Features of SARS-CoV-2–Positive and –Negative Respiratory Tract Infections**

Jaakko Ahti, Laura Toivonen, Helena Ollila, Lauri Ivaska, Krista Salo-Tuominen, Tytti Vuorinen, Johanna Lempainen, and Ville Peltola

**Supplementary Materials**

**Supplementary Methods**

**Supplementary Figure 1. Flowchart of Participants and Households in the Study**

**Supplementary Figure 2. Flowchart of Clusters Included in the Analysis**

**Supplementary Figure 3. Circulation of Respiratory Viruses in the Study Region**

**Supplementary Table 1. Emergence of Omicron BA.1. and BA.2 Variants in the Study Region**

**Supplementary Table 2. Prevalence and Duration of Symptoms of Respiratory Infections Positive and Negative for SARS-CoV-2**

**Supplementary Table 3. Comparison of Symptoms Between Children and Adults in SARS-CoV-2–Positive and –Negative Infections**

**Supplementary Table 4. Mean Weekly Number of Responses to REDCap Questionnaires**

**SUPPLEMENTARY METHODS**

**Follow-up**

Weekly REDCap questionnaires of respiratory infections covered all household members. In detail, participants were asked to fill in the dates of symptoms of respiratory infection (fever, cough, rhinorrhea, sore throat, fatigue, shortness of breath, headache, loss of smell or taste, muscle pain, diarrhea, vomiting, and other symptoms), physician visits with diagnosis and treatment, date and results of nasopharyngeal specimens tested for SARS-CoV-2 by PCR in the laboratory, and date and results of home antigen tests for SARS-CoV-2 for each household member.

Surveillance data on circulating respiratory viruses in the study region was obtained from the Department of Clinical Microbiology at the Turku University Hospital.

**National COVID-19 Vaccination Policies, SARS-CoV-2 Testing Strategies, and Restrictive Measures in Finland**

COVID-19 vaccinations were started for adults in Finland at the turn of 2020–2021 using mostly mRNA vaccines and more rarely adenovirus vector vaccines. Vaccinations of adolescents aged 12–17 years and children aged 5–11 years were started in August and December 2021, respectively, with two doses of mRNA vaccine with an interval of 6-12 weeks between doses.

According to national guidelines, adults and children with symptoms compatible with COVID-19, and also those exposed to COVID-19 were tested for SARS-CoV-2 until late fall 2021, when testing of asymptomatic low-risk persons was discontinued. The recommendation to test all symptomatic persons continued throughout the study period in Finland. When home antigen tests became widely available from January 2022 onward, PCR testing was targeted to groups at high risk of severe COVID-19, healthcare personnel, persons with severe symptoms, and working-age persons without an option to work from home (due to infectious disease allowance).

In Finland, various non-pharmaceutical measures were implemented between 2020 and 2022 to prevent the spread of COVID-19. From March 2020 to May 2020, November 2020 to June 2021, and September 2021 to January 2022, gatherings were restricted to a varying degree depending on the region and time, with limits ranging from 10 to 500 people per gathering. Rules for outdoor gatherings were more permissible. Restrictions were lifted when the numbers of COVID-19 cases were low but re-implemented when the incidence rate increased. The use of face masks in public was recommended starting from November 2020. During the study period, lower secondary schools (grades 7-9, 12-16-year-olds) and upper secondary schools (15-19-year-olds) adopted remote learning from 8th March 2021 to 11th April 2021 in Southwest Finland. However, primary schools (grades 1-6, 6-13-year-olds) continued operating normally during this time. In the period between autumn 2020 and spring 2021, schools frequently resorted to remote learning for a period ranging from one to three weeks in response to the incidence of SARS-CoV-2 cases in the school or a high prevalence of the local epidemic. These decisions were made at the discretion of individual schools. Schools operated normally from autumn 2021 onward, apart from the facemask recommendation that was authorized at the end of 2021 and lifted in spring 2022


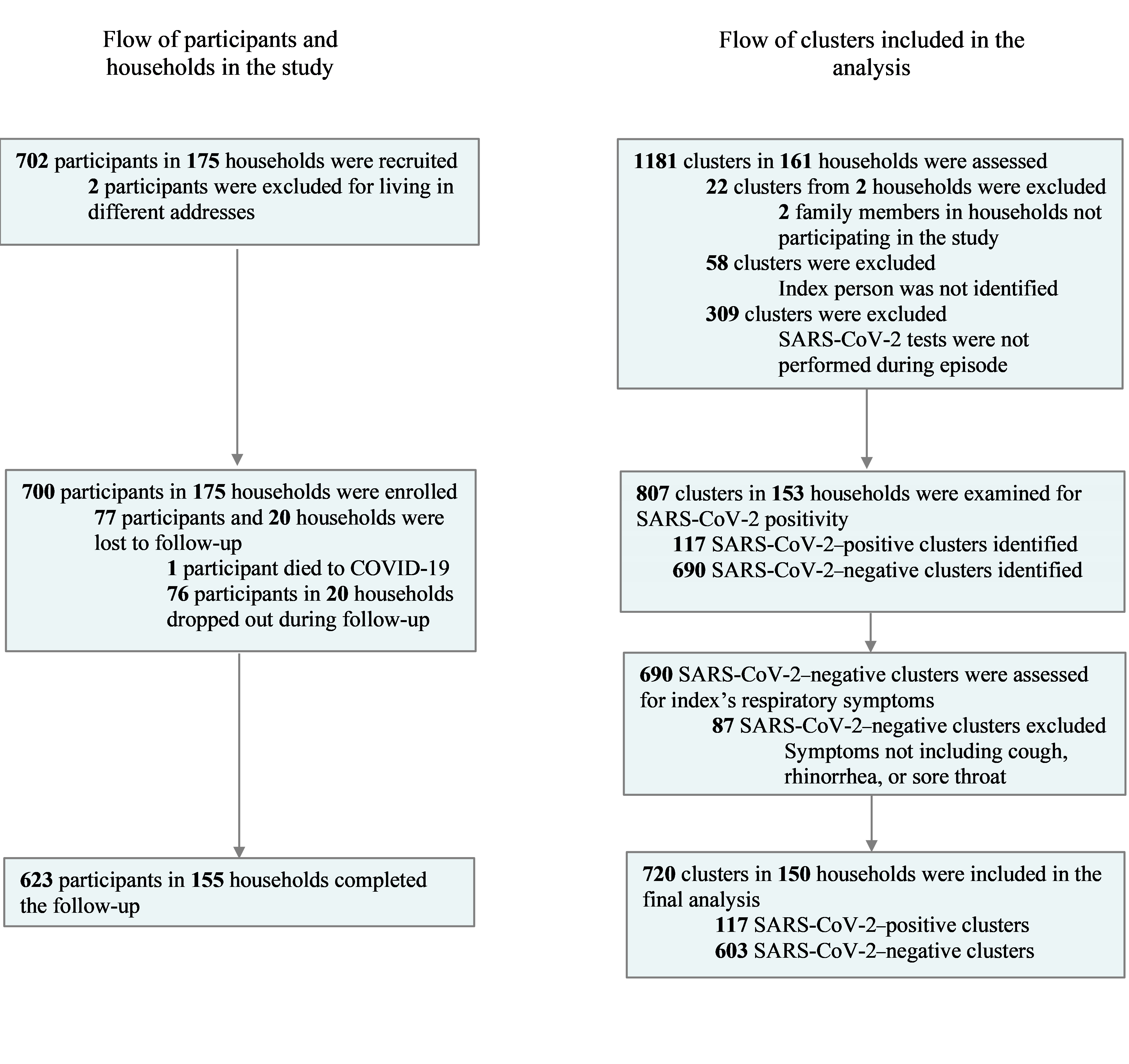


**Supplementary Figure 1. Flowchart of Participants and Households in the Study**


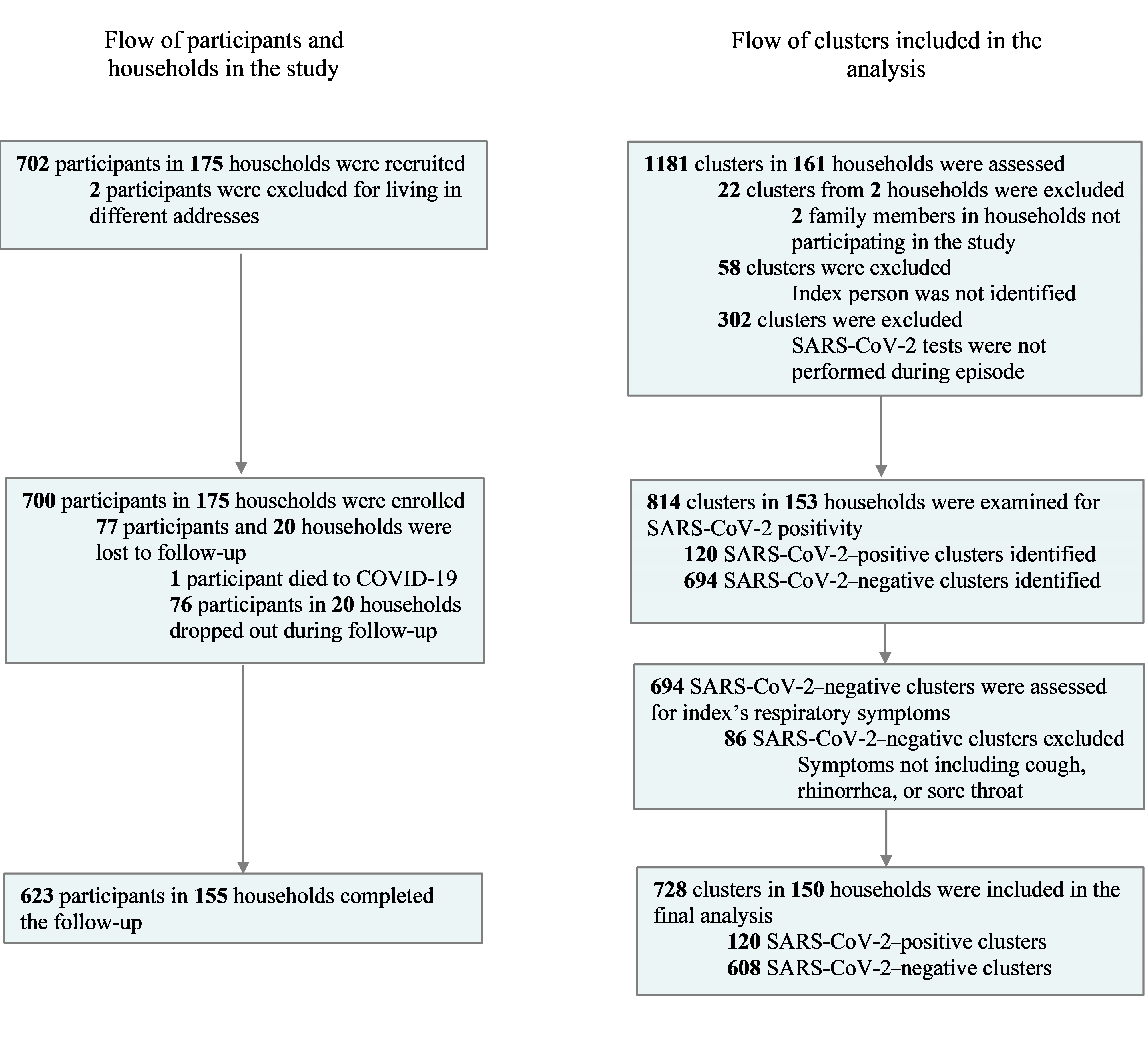


**Supplementary Figure 2. Flowchart of Clusters Included in the Analysis**

**
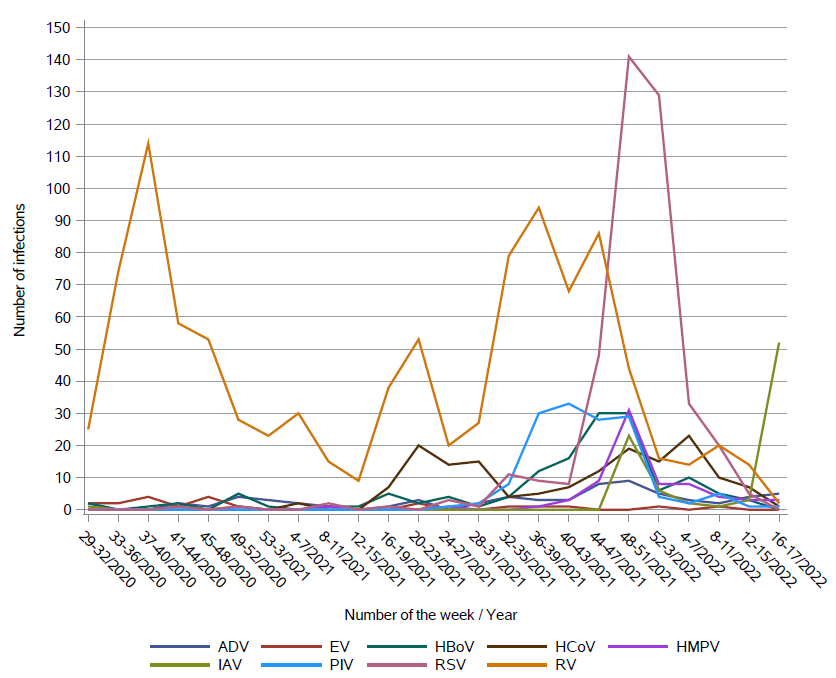
**

**Supplementary Figure 3. Circulation of Respiratory Viruses in the Study Region**

ADV, adenovirus; EV, enterovirus; HBoV, human bocavirus; HCoV, human coronavirus; HMPV, human metapneumovirus; IAV, influenza A virus; PIV, parainfluenza virus; RSV, respiratory syncytial virus; RV, rhinovirus. HCoV includes human coronaviruses OC43, NL63, and 229E. Influenza B viruses (n = 3) are not shown.

**Supplementary Table 1**. **Emergence of Omicron BA.1. and BA.2 Variants in the Study Region^a^**

| Week/Year | SARS-CoV-2–Positive Tests, No. | Omicron BA.1, n (%) | Omicron BA.2, n (%) |
| --- | --- | --- | --- |
| 49/2021 | 582 | 0 (0) |  |
| 50/2021 | 715 | 35 (4.9) |  |
| 51/2021 | 978 | 413 (42.2) |  |
| 52/2021 | 1831 | 1387 (75.8) |  |
| 1/2022 | 2954 | 2626 (88.9) |  |
| 2/2022 | 3719 | 3456 (92.9) |  |
| 3/2022 | 2911 | 2777 (95.4) |  |
| 4/2022 | 1624 | 1532 (94.3) |  |
| 5/2022 | 874 |  | 39 (4.5) |
| 6/2022 | 2438 |  | 185 (7.6) |
| 7/2022 | 2527 |  | 248 (9.8) |
| 8/2022 | 2083 |  | 372 (17.9) |
| 9/2022 | 2538 |  | 1005 (39.6) |
| 10/2022 | 2836 |  | 1509 (53.2) |
| 11/2022 | 3032 |  | 1975 (65.1) |
| 12/2022 | 2769 |  | 2163 (78.1) |
| 13/2022 | 1806 |  | 1468 (81.3) |
| 14/2022 | 414 |  | 366 (88.4) |
| 15/2022 | 1031 |  | 966 (93.7) |

^a^Data obtained from the Department of Clinical Microbiology, Turku University Hospital.

**Supplementary Table 2.** **Prevalence and Duration of Symptoms of Respiratory Infections Positive and Negative for SARS-CoV-2**

| Symptoms | Total,  n = 1332 | SARS-CoV-2–Positive,  n = 269^a^ | SARS-CoV-2–Negative,  n = 1063 | OR (95% CI)^b^ | *P* Value^c^ |
| --- | --- | --- | --- | --- | --- |
| Fever  Prevalence, n (%)  Duration, d; median (IQR) | 329 (25)  2 (2-3) | 134 (51)  3 (2-4) | 195 (18)  2 (2-3) | 6.57 (4.63-9.33) | <0.0001  0.047 |
| Cough  Prevalence, n (%)  Duration, d; median (IQR) | 624 (47)  5 (3-7) | 174 (66)  4.5 (3-8) | 450 (42)  5 (3-7) | 2.70 (1.98-3.67) | <0.0001  0.87 |
| Rhinorrhea  Prevalence, n (%)  Duration, d; median (IQR) | 949 (72)  5 (4-7) | 179 (68)  5 (3-7) | 770 (72)  5 (4-7) | 0.84 (0.62-1.15) | 0.28  0.75 |
| Sore throat  Prevalence, n (%)  Duration, d; median (IQR) | 888 (67)  3 (3-5) | 176 (67)  4 (3-5) | 712 (67)  3 (2-5) | 1.08 (0.79-1.48) | 0.63  0.045 |

Abbreviations: CI, confidence interval; IQR, interquartile range; OR, odds ratio; SARS-CoV-2, severe acute respiratory syndrome coronavirus 2.

^a^Asymptomatic SARS-CoV-2 infections (n = 7) were excluded.

^b^SARS-CoV-2–positive infections compared to SARS-CoV-2–negative infections.

^c^Symptom prevalences were compared using mixed-effects logistic regression and durations with a linear mixed model.

**Supplementary Table 3. Comparison of Symptoms Between Children and Adults in SARS-CoV-2–Positive and –Negative Infections**

| Symptoms | SARS-CoV-2–Positive | | | | SARS-CoV-2–Negative | | | |  |
| --- | --- | --- | --- | --- | --- | --- | --- | --- | --- |
|  | Child  n = 149^a^ | Adult  n = 120 | OR  (95% CI)^b^ | *P* Value^c^ | Child  n = 656 | Adult  n = 407 | OR  (95% CI)^b^ | *P* Value^c^ | |
| Fever  Prevalence, n (%)  Duration, d; median (IQR) | 73 (51)  3 (2-3) | 61 (51)  3 (2-4) | 1.11  (0.62-1.98) | 0.71  0.24 | 135 (21)  2 (2-3) | 60 (15)  2 (2-3) | 1.51  (1.04-2.18) | 0.029  0.11 | |
| Cough  Prevalence, n (%)  Duration, d; median (IQR) | 83 (58)  4 (3-7) | 91 (76)  5 (4-9) | 0.43  (0.23-0.80) | 0.013  0.56 | 294 (45)  5 (3-7) | 156 (38)  5 (4-8) | 1.25  (0.95-1.64) | 0.11  0.098 | |
| Rhinorrhea  Prevalence, n (%)  Duration, d; median (IQR) | 83 (58)  5 (3-6.5) | 96 (80)  5 (3-7.5) | 0.35  (0.18-0.65) | 0.0036  0.79 | 471 (72)  5 (4-7) | 299 (73)  5 (4-7) | 0.92  (0.68-1.25) | 0.61  0.62 | |
| Sore throat  Prevalence, n (%)  Duration, d; median (IQR) | 93 (65)  4 (3-6) | 83 (69)  3 (3-5) | 0.80  (0.43-1.49) | 0.44  0.26 | 445 (68)  3 (3-5) | 267 (66)  3 (2-5) | 1.12  (0.83-1.53) | 0.46  0.57 | |

Abbreviations: CI, confidence interval; IQR, interquartile range; OR, odds ratio; SARS-CoV-2, severe acute respiratory syndrome coronavirus 2.

^a^Asymptomatic SARS-CoV-2 infections (n = 7) were excluded.

^b^Children compared to adults.

^c^Symptom prevalences were compared using mixed-effects logistic regression and durations with a linear mixed model.

**Supplementary Table 4. Mean Weekly Number of Responses to REDCap Questionnaires**

| Quarter/Year | Responses,  Mean No. / Week |
| --- | --- |
| Q4/2020 | 117 |
| Q1/2021 | 118 |
| Q2/2021 | 115 |
| Q3/2021 | 110 |
| Q4/2021 | 102 |
| Q1/2022 | 100 |
